# Problem drug use the public health imperative: what some of the literature says

**DOI:** 10.1186/1747-597X-4-21

**Published:** 2009-12-16

**Authors:** Gez Bevan

**Affiliations:** 1 University of Sunderland, Faculty of Applied Sciences, Chester Rd, Sunderland, SR1 3SD, UK

## Abstract

**Background:**

With more than 200,000 problem drug users is contact with structured treatment services in England the public health imperative behind drug treatment is great. Problem drug use for many is a chronic and relapsing condition, where "cure" is often neither a reasonable or appropriate expectation and it can further be argued that in these circumstances problem drug use is no different from any number of chronic and enduring health conditions that are managed in the health care system and therefore should be conceptualised as such.

**Discussion:**

A public health approach to drug treatment emphasises the need for drug users in or accessing treatment, to reduce their harmful drug use, reduce drug use related risks such as sepsis and overdose and stay alive for longer. However a public health perspective in relation to problem drug use isn't always either apparent or readily understood and to that end there is still a significant need to continue the arguments and debate that treatment and interventions for problem and dependent drug users need to extend beyond an individualistic approach. For the purposes of discussion in this article public and population health will be used interchangeably.

**Summary:**

A recognition and acceptance that a public and population health approach to the management of problem drug users is sound public health policy also then requires a long term commitment in terms of staffing and resources where service delivery mirrors that of chronic condition management.

## Background

The history to substance misuse treatment and interventions has cycled through a variety of ideologically driven approaches and perspectives covering medicalisation, treatment, decriminalisation, the overt use of the criminal justice as quasi treatment as well as more accepting positions such as harm minimisation and harm reduction. The purpose of this article is to continue the debate which makes a case for a public and population health imperative behind substance misuse treatment, highlighting the chronic and enduring nature of substance misuse for many along with the increase in morbidity and mortality amongst drug users relative to their non drug user contemporaries, as well as the evidence base which underpins effective treatment modalities.

## Discussion

### A Public Health Perspective

Figures from the National Treatment Agency for Substance Misuse (NTA) report that 207,580 individuals, aged 18 and over, were recorded as being in contact with structured drug treatment services in England for the counting period 2008/09, this included General Practitioners (GPs) providing structured treatments, that would typically, but not exclusively, be prescribing modalities; this is a rise on the 2007/8 figures of 202,666 individuals recorded as being in contact with structured drug treatment services in England during 2007/8 which in turn is an increase on the 2006/7 numbers of 195,464 of individuals recorded as being in contact with specialist drug treatment services and a further increase from the 2005/6 data of 177,055 [1]. It is true that processes within the NTA and the National Drug Treatment Monitoring System (NDTMS) have changed over this time period which has meant that some of the specifics of the data set and its definitions have altered, for example the inclusion criteria changing from specialist services to structured treatment services as well as more robust data collecting systems; these changes could in themselves generate discussion and debate around methods, methodology, data verification and veracity. However that said these figures demonstrate that problem drug use is not an insignificant issue and moreover that since these figures represent only those in contact with structured drug treatment services the overall prevalence of problem drug users can be expected to be substantially higher; on the basis of these statistics alone it is clear that the population health impact of problem drug use cannot be doubted.

So what exactly is a public health perspective in relation to problem drug use? The primary focus is one of reducing harm amongst identified populations, and arguably the best way of achieving this is by minimising risk. Harm reduction can broadly be argued to be a range of policies, programmes and interventions aimed at reducing the harm caused by problem drug use [2,3] A more precise definition from The International Harm Reduction Association is, "to reduce the health, social and economic harms associated with the use of psychoactive substances" [4]. Developing out of the rise of the Human Immunodeficiency Virus (HIV) in 1980s, harm reduction became a significant public health force putting the population health impact and consequences of problem drug use on the political agenda [5,6]

### Problematic Drug Use

A problem drug user can be defined as someone who experiences a range of unwanted and negative consequences as a result of their drug use [7] namely social, psychological, physical or legal problems related to regular or excessive consumption, intoxication and/or dependence. The NTA describe problematic drug use as being a chronic and relapsing condition [8] often taking several attempts to achieve stability or abstinence, The World Health Organisation (WHO) concur that problem drug use is best regarded as a chronic behavioural disorder, where controlling or stopping the problem drug use frequently takes many attempts, and moreover that relapse is common [9] This is echoed further in that problem drug use, for many, results in a repeated cycle of treatment, relapse and recovery that might last for decades [10]

### Chronic & Relapsing

The Royal Pharmaceutical Society in the UK have long argued that treatment for problem drug use is likely to be punctuated by times of crisis or relapse [11] and rather than seeing these as a failure of the patient or programme, recognising that problem drug use is frequently a chronic and relapsing condition is a far more useful way of explaining events. Often characterised as a career that typically lasts upwards of 10 years, problem drug use is marked by oscillations between relapse and recovery [12] These two concepts of relapse and recovery are both pivotal to an understanding and application of substance misuse treatment [13,14] Covering several domains of behaviour, relapse prevention aims to deal with obstructive and negative beliefs which lead to repeat behaviours. The domains covered include; negative emotions, negative physical states, urges & cravings and dealing with inter-personal conflicts. Effective relapse prevention strategies can be particularly good at addressing relatively and somewhat disruptively short treatment episodes, they also provide an opportunity to better understand why relapse occurs and what preventative measures can be put in place, thus enabling clients to move forward.

The use of Recovery Management Checkups (RMCs) were explored and found to be invaluable in enabling those who have relapsed to be able to re-access treatment the net result being that those utilising the RMCs re-entered treatment sooner after dropping out, were in receipt of more treatment and the time between relapse and treatment re-entry was shortened significantly [15]. A conclusion reached by some however is that for the majority of chronic problem drug users the long term outcomes are not favourable and that growing out of their substance misuse simply does not apply [16]

The Royal College of General Practitioners in the UK also regard substance misuse as being a chronic and relapsing condition [17] and as such they make the important distinction that substance misuse should, conceptually at least, be no different to any number of chronic conditions managed in Primary Care. Chronic health conditions, across the spectrum of physical and mental health are often united by the single feature that cure is not an available option [18,19] however effective management of the condition is achievable; ameliorating symptoms as necessary and enabling patients and service users to recognise what might trigger episodes of relapse is paramount to an understanding of problem drug use, in much the same way as other chronic conditions such as diabetes, heart disease, multiple sclerosis, Parkinson's disease and enduring mental illnesses are understood

Long term conditions are further characterised by poor compliance and limited adherence to treatment plans and advice, [20-24] Moreover in almost all the literature reviewed there was an acknowledgement that the reasons precipitating non-compliance were complex and varied and not necessarily linked to lack of knowledge or the ability to understand; concordance with treatment regimes and advice requires both the client and the health care professional to have the same perspectives and negotiated goals.

The concept of compliance has been explored further [25] and the observation made that for many clinicians their definition of compliance is the extent to which the patient or client takes the medication as it is prescribed, whilst well defined this definition is also very restrictive and concordance is probably a more useful term and is suggestive of the need for clinicians to form a more effective therapeutic alliance with service users to facilitate them in adhering to treatment plans rather than blaming them when they don't [26-28] In the literature reviewed they all make the fundamental point that compliance is best achieved through a working relationship which is based upon a shared understanding of the issues, how these issues can best be addressed and placing the non compliance within a context of understanding why people default from optimal treatment rather than coming from a moral perspective which is that they have in some way failed. Compliance is frequently value laden where compliance with advice is considered good and non-compliance is seen as bad, this fundamental difference in moral understanding seems to do little for developing an effective therapeutic alliance. A starting point which emanates from professionally ingrained attitudes and beliefs seems always to be fraught with dissonance.

Lack of compliance or dis-engagement from treatment has little to do with the specifics of being a drug user, moreover this is better explained by the complexities of living with a chronic (and relapsing) condition and how individuals make choices and endeavour to exercise some control in situations and circumstances where this balance is otherwise skewed. Out with of the realms of addiction probably the very best example of non compliance affecting the general population is that of non completion of courses of antibiotics [29,30] with an interesting related observation being made that non-compliance or non-adherence with prescribed treatment might be more easily explained by study outcomes which identify that patients cease to take the medication because they feel better or because the prescribing regime was too onerous or complicated to fit in with a normalised life style [31].

The parallels with opiate replacement prescribing are evident; being committed to a treatment modality might also require frequent attendance at a community pharmacy or treatment service in order to receive the prescribed treatment and in turn this requires motivation to attend in the belief that the outcome will be beneficial. The issue of being sufficiently motivated to engage in substance misuse treatments is well documented [32-34] and most notably by Sellman [35] where there is an overt reference made to the dilemmas and limitations associated with being required to be "sufficiently motivated" so as to engage in treatment. The requirement to demonstrate motivation seems also to stem from a moral perspective that access to health care and services is frequently a reflection of being deserving or undeserving and that "tests" of commitment will in someway sift out those who are not serious, committed or motivated to change. One concern however is that having to demonstrate motivation in order to access treatment brings substance misuse treatment back to the individualistic level and does little to reduce the public and population health risks associated with drug use, and injecting drug use in particular.

### Problem Drug Use Treatments

Problem drug use treatment can typically be defined as interventions that directly address or ameliorate the negative effects of problematic drug use and cover a broad spectrum, from pharmacological interventions such as substitute prescribing and detoxification, through to psycho-social approaches including models of counselling and behaviour modification namely Cognitive Behavioural Therapy, Transtheoretical Model (cycle of change) & Motivational Interviewing as well as specific harm reduction interventions; needle exchange, Hepatitis screening and vaccination programmes. As important as the treatment and care is which directly addresses the problem drug use, focusing exclusively on these treatment interventions results in other drug use related issues such as sexual health, pregnancy, mental and psychological health falling outside the remit; the population's health benefits most from being addressed and managed from an inclusive i.e. public health and harm reductionist approach.

Evidence supporting the benefits and gains that are achieved through availability of treatment, even if abstinence is not achieved, is demonstrated through the literature and that being punishing or excluding does nothing to reduce substance use, moreover it only creates situations where the level of harm and risk are escalated [36] The benefits of unhindered and probably more controversial unconditional access to treatment and health care for problem drug users cannot be doubted. Patients retained in methadone treatment within primary care improved on a range of harm reduction measures including HIV risk-taking behaviour as well as in their physical and psychological health [37] with others concluding that treatment duration was associated with positive improvements in primary drug use and that being in treatment was associated with a reduction of non fatal overdose that was directly related to a reduction in high risk practices, demonstrating that treatment can change behaviour [38,39] Treatment also substantially increases the likelihood of abstinence from illicit drugs, but even where abstinence is not achieved there can still be substantial reductions in frequency of use of heroin, non-prescribed methadone, benzodiazepines, and crack cocaine; associated Injecting and shared use of injecting equipment are also reduced [40,41] further supporting the argument that the health of populations can improve significantly when associated drug use harms are reduced.

### Substitute Prescribing

Substitute prescribing, as a harm reduction strategy, is supported by the wealth of evidence that appropriately and safely prescribed higher doses of long acting opioids such as methadone are more effective in both reducing illicit opiates use, and in retaining problem drug users in treatment services for longer. Predictors of retention in drug treatment have demonstrated over time to be inextricably linked to therapeutic doses of methadone that are higher than 60 mg per day, [42-47]

Allied to the retention in treatment is the reduction in illicit opiate use, and with it a reduction in the attendant risks of overdose, sepsis and exposure to communicable disease, again there is significant evidence, over almost 2 decades that higher methadone doses assist drug users in using less illicit opiates [48-53] Adopting a harm reduction philosophy towards drug treatment prescribing, where illicit or non prescribed psychoactive drug use in addition to prescribed treatments is acknowledged as being likely to happen, rather than pursuing an enforced abstinence as the eligibility criteria is further demonstrated to retain clients in treatment, whilst at the same time reducing their additional drug use [54-57]

### Preventing the transmission of Communicable Disease

The benefits to the health of populations of preventing the transmission of communicable diseases, particularly blood borne virus, is well documented and in particular the introduction of harm reduction interventions during 1980's is argued to account for the low prevalence of HIV amongst injecting drug users in England seen during 1990's where the incidence of HIV prevalence amongst injecting drug users declined from 5.9% in 1990 to 0.6% in 1996 [58]. However, in contrast to the figures in England there were exceptionally high rates of HIV infection amongst Injecting Drug Users in Scotland, particularly Edinburgh [59-62] with prevalence rates of 50% or more [63,64] the lack of and in some case prohibition of needle and syringe exchange programmes was not insignificant in resulting in these numbers.

The expansion of needle exchange programmes have been seen to be the single most effective harm reduction intervention in keeping the transmission levels of HIV amongst injecting drug users in the England at their consistently low levels [65] Whilst not an invitation for complacency it does add weight to the continued argument that as an intervention in reducing transmission of HIV amongst injecting drug users needle exchange does work, regardless of the arguments about the legality of heroin use and does offer a differential perspective, one of keeping the health benefits of treatment separate from those of the Criminal Justice System.

Hepatitis C prevalence amongst Injecting Drug Users is extremely high [66-70] Documented prevalence of Hepatitis C amongst injecting drug users being between 50-90% and sharing injecting equipment and paraphernalia is identified as the behaviour more likely to result in transmission than any other risk taking behaviour [71-75] Unlike with Hepatitis C there is however a recognised method of preventing the spread of Hepatitis A & B through effective and safe vaccination [76-79] The ease with which transmission of these two viruses can occur adds weight to the argument that a population health approach is both essential and morally defensible, not least of all the rapid transmission of Hepatitis A in close social and domestic circumstances and the ease with which Hepatitis B is transmitted both sexually and in utero [80,81]

The UK's current Hepatitis B vaccination strategy has been based upon the selective targeting of high risk groups [82-84] and despite a rising prevalence amongst injecting drug users and in particular those within prisons this continues to be pursued [85,86] and whilst it is the case that the UK does not experience the high endemicity and chronic carrier numbers present elsewhere [87-89] there is a wealth of evidence that the recommendations made by the WHO in 1992, namely that Hepatitis B should be incorporated into national vaccination programme globally, was not without foundation since vaccination works and is feasible to introduce [90,91] Moreover it also goes some way to address the low risk perception and knowledge which exists, particularly amongst young adults and most worryingly amongst injecting drug users where there was often a dissonance between their perception or belief of their hepatitis status and laboratory virology [92-96].

It continues to be a source of debate that there is a distorted logic which prevails where sections of the population are offered effective protection via vaccination only once they are exposed to the risks of Hepatitis B and there then is a significant amount of effort required to redeem the situation and encourage uptake of vaccination.

### Reducing Drug Related Deaths

Morbidity and mortality frequently go hand in hand for problem drug users, particularly injecting drug users [97] Significant evidence exists that drug users are at a greater risk of premature death than their non drug using contemporaries [98-101] with some estimates putting the differential 13-17 times greater than non drug users [102,103] In terms of reducing drug related deaths, substitute prescribing and in particular methadone maintenance is demonstrated to be a protective factor against premature death. In short, longer term opiate replacement therapy keeps problem drug users in treatment for longer and keeps those who are in treatment alive. Most importantly however is the evidence that early or involuntary cessation of methadone treatment, particularly due to a breach of the treatment service rules, results in higher mortality rates compared with those retained in treatment [104-106]

The benefits of treatment, in reducing non-fatal overdose, were also evident from the Australian Treatment Outcome Study (ATOS) [107] with non fatal overdose rates over 12 months falling from 24% to 12%, methadone maintenance treatment demonstrated a significant reduction in overdose, from 22% to 4%. This reduction in overdose was related to a reduction in the frequency of drug use and lower rates of injecting. Unsurprisingly, as the discussion has already demonstrated, more treatment episodes suggestive of interrupted treatment and episodes that are multiple but reduced in overall length and typically punctuated by untimely disruption eg imprisonment, results in an increased risk of overdose. Sustained and seamless drug treatment has significant benefits in reducing premature deaths amongst problem drug users.

## Summary

The primary focus of a public health approach to substance misuse is that of reducing harm amongst identified populations

Substance misuse for many is deemed to be a chronic condition often further characterised by poor compliance and limited adherence to treatment plans and advice

Morbidity and mortality are high with premature deaths a significant cause for concern

The evidence base in support of substitute prescribing reports consistently that doses in excess of 60 mg per day have better efficacy than lower (sub optimal doses)

## Conclusion

The Public Health imperative behind substance misuse treatments is best demonstrated when the rationale for drug treatment accepts that for many who receive treatment it is a chronic and frequently relapsing condition. The purpose behind treatment needs to move away from an individualistic approach to one which recognises drug users exist in many and varied relationships and as part of several communities, thus the health of populations becomes a pivotal part of the treatment rationale. A public health imperative for the delivery of drug treatments can be seen to be effective in minimising the harm associated with drug use, both for the individual and the communities they reside within. The use of needle exchanges in reducing some blood borne virus transmission is clearly demonstrated, clients retained in treatment for longer have more favourable treatment outcomes, both drug use and risk taking are reduced when prescribing policies for opiate replacement are not contingent upon abstinence of other substances and where appropriately prescribed doses of methadone are in excess of 60 mg per day.

The reduction in drug related deaths, as a result of the reduced frequency of accidental overdoses, and the wider population health gains when treatment and interventions are flexibly delivered is further evidence that the health of populations can be improved with public health based strategies for substance misuse. Whilst concordance with treatment can at times be inconsistent, evidence from the literature demonstrates that this is not the exclusive preserve of problem drug users; it is however the very nature of an enduring condition which means compliance with treatment is often sub-optimal.

People change their behaviour in stages; few people make radical life changing moves that take them from one extreme to the other in a short period of time. This incremental approach to behaviour change is both more sustainable and manageable and more importantly views relapse as part of the stages rather than being a value laden judgement on the individual who is deemed to have "failed". Limiting the cascade effect of drug related harm within populations needs to be the philosophy adopted and engaging in punitive battles with individuals serves no useful purpose; moreover it actually serves only to use individuals as scapegoats when the health of populations is the responsibility of many and not the fault of the few.

To cure problem drug users is frequently not a reasonable approach to take, and an over emphasis on moving people on, through and out of treatment frequently results in the revolving door phenomena; these are however both politically challenging and contentious issues. Substitute prescribing in particular can have a long term impact upon service provision and capacity. When there is a real understanding and acceptance that some in receipt of substitute prescribing do best when this is long term and at a maintenance dose, what invariably follows has to be an acceptance that substitute prescribing for some might cover 5, 7, 10 or more years and this needs not only to be accepted as being clinically appropriate but also likely to occur and thus resourced appropriately. Only when individuals who use drugs in a problematic manner are viewed as being part of a wider society and members of dynamic social groups with families, friends and acquaintances do the public health implications and ramifications become obvious.

In the light of the evidence it is clear that problem drug use is frequently a chronic and relapsing condition, requiring ongoing management over a number of years or decades, where the consequences go beyond the individual and is a condition that can and does result in premature and avoidable deaths. There is a pressing need that public health principles should in fact be the foundation of all drug treatment interventions, and that investment in drug treatment is sound public health policy.

## Abbreviations

ATOS: Australian Treatment Outcome Study; NTA: National Treatment Agency for Substance Misuse; HIV: Human Immunodeficiency Virus; WHO: World Health Organisation.

## Competing interests

The author declare that she has no competing interests.

## Authors' contributions

The article was organised, structured and written entirely by GB.
